# Selective Activation of Alternative *MYC* Core Promoters by Wnt-Responsive Enhancers

**DOI:** 10.3390/genes9060270

**Published:** 2018-05-23

**Authors:** Jorge A. Bardales, Evin Wieser, Hideya Kawaji, Yasuhiro Murakawa, Xavier Darzacq

**Affiliations:** 1 Department of Molecular and Cell Biology, University of California, Berkeley, CA 94720, USA; jorgebardales@berkeley.edu (J.A.B.); ewieser94@gmail.com (E.W.); 2 Biophysics Graduate Group, University of California, Berkeley, CA 94720, USA; 3 Division of Genomic Technologies, RIKEN Center for Life Science Technologies, Yokohama 230-0045, Japan; kawaji@gsc.riken.jp (H.K.); yasuhiro.murakawa@riken.jp (Y.M.); 4 RIKEN Preventive Medicine and Diagnosis Innovation Program, Yokohama 230-0045, Japan; 5 Preventive Medicine and Applied Genomics Unit, RIKEN Advanced Center for Computing and Communication, Yokohama 230-0045, Japan; 6 Li Ka Shing Center for Biomedical and Health Sciences, CIRM Center of Excellence, University of California, Berkeley, CA 94720, USA

**Keywords:** alternative promoters, enhancer-promoter specificity, *MYC*, Wnt-responsive enhancers

## Abstract

In Metazoans, transcription of most genes is driven by the use of multiple alternative promoters. Although the precise regulation of alternative promoters is important for proper gene expression, the mechanisms that mediates their differential utilization remains unclear. Here, we investigate how the two alternative promoters (P1, P2) that drive *MYC* expression are regulated. We find that P1 and P2 can be differentially regulated across cell-types and that their selective usage is largely mediated by distal regulatory sequences. Moreover, we show that in colon carcinoma cells, Wnt-responsive enhancers preferentially upregulate transcription from the P1 promoter using reporter assays and in the context of the endogenous Wnt induction. In addition, multiple enhancer deletions using CRISPR/Cas9 corroborate the regulatory specificity of P1. Finally, we show that preferential activation between Wnt-responsive enhancers and the P1 promoter is influenced by the distinct core promoter elements that are present in the *MYC* promoters. Taken together, our results provide new insight into how enhancers can specifically target alternative promoters and suggest that formation of these selective interactions could allow more precise combinatorial regulation of transcription initiation.

## 1. Introduction

RNA Polymerase II-dependent transcription initiation is a complex process that must be precisely regulated for the proper generation of spatio-temporal patterns of gene expression. The core promoter plays an essential function in integrating multiple transcriptional signals, including tissue-specific enhancer mediated activation to modulate the rate of transcription initiation [1,2,3]. The core promoter can be defined as a short stretch of DNA surrounding the transcription start site (TSS) that potentiates directional transcription initiation. Core promoters are diverse in their composition of *cis*-control DNA and can contain multiple elements like transcription factor IIB (TFIIB) recognition sites (BRE^u^ and BRE^d^), TATA box binding sites, initiator sequences (INR), motif ten elements (MTE) and downstream core promoter elements (DPE) [3,4]. These diverse core promoter elements can serve two roles in which they can modulate the basal activity of core promoters [5,6], and they can regulate the core promoter capacity to be activated by specific enhancers and other distal *cis*-regulatory sequences [7,8,9,10]. 

Numerous reports have shown that in metazoans, most genes are under the control of multiple alternative promoters [11,12,13,14]. In fact, the most recent report from the FANTOM consortium [15], which characterized more than 900 human cell samples by using Cap Analysis of Gene Expression (CAGE) found that genes, on average, can utilize 4 robust TSSs [15]. Interestingly, alternative promoters have been found to be differentially utilized across different tissues [11,16], during development and upon the presence of specific stimuli [11,17,18], suggesting that fine tuning of alternative promoters may be important for proper gene expression. Importantly, misregulation of alternative core promoter usage has been found to be associated with pathological states including cancer and neurodegenerative disorders [19,20,21]. Although some evidence suggests that promoter usage is largely dependent on the epigenetic landscape of chromatin [22,23,24,25] and the presence of proximal transcription factor binding sites [26,27,28], the specific mechanisms that could regulate alternative promoter usage remains poorly understood.

*MYC* is a key transcription factor that has been shown to regulate the expression of thousands of genes involved in a wide-range of key cellular processes including cell growth, proliferation, and metabolism [29,30]. Given the important physiological role of *MYC*, it is not surprising then that *MYC* expression needs to be tightly regulated, especially at the transcription level [31,32]. Two alternative tandem promoters, P1 and P2, drive the expression of *MYC* in most cells [32]. These two *MYC* promoters integrate multiple regulatory signals, including hundreds of tissue-specific enhancers [33,34,35,36], and promoter proximal regulatory sequences [18,25,37,38,39] to precisely regulate *MYC* transcription. In this report, by analyzing CAGE data, we find that the two *MYC* promoters can be differentially regulated across different human cell samples. Importantly, using synthetic reporter assays, we found that the activity of the *MYC* promoters embedded in transgenic constructs does not recapitulate the differential promoter usage observed in the context of the endogenous *MYC* locus, suggesting that promoter usage is likely to be regulated by distal regulatory elements. This led us to test in colon carcinoma cells if distal *MYC* enhancers that are responsive to Wnt signaling could differentially regulate the endogenous activity of the two promoters. We observed that Wnt-responsive enhancers preferentially activate the P1 promoter upon Wnt induction. In addition, by using CRISPR/Cas9, we confirmed that enhancer deletion preferentially downregulates the activity of the P1 promoter. Finally, we demonstrated that preferential activation of the P1 promoter is mediated by distinct promoter elements associated with P1 that differ from P2. Taken together, these results suggest that alternative promoters can mediate cell-type selective transcription regulation by facilitating selective interactions with distal and proximal Wnt-responsive enhancers.

## 2. Materials and Methods

### 2.1. CAGE Data Analysis

The CAGE profile of the *MYC* locus was obtained from the FANTOM5 database (http://fantom.gsc.riken.jp) [40]. We accessed the Zenbu data viewer of the human promoterome (https://bit.ly/2JmEnVR) [41] to obtain the total number of reads from the *MYC* P1 and P2 promoters in a 11 bp window around the TSS. A threshold of 0.1 cpm. was set for each promoter before we determined the frequency distribution of the log_2_ (P2/P1). Similarly, for the time courses, we accessed the Zenbu data viewer human time course (https://bit.ly/2HpU9Pd) [41], to obtain the number of reads for all the differentiation time courses which were used to calculate the log_2_ (P2/P1) promoter usage ratio at the different time points. The protocols describing the time courses and specific cell lines used are described by Arner et al. [42].

### 2.2. Cell Culture

All cell lines used in this study were cultured in accordance with ATCC and Riken Cell bank guidelines in their suggested media supplemented with 10% fetal bovine serum in a humidified incubator at 5% CO_2_. For HCT-116 cells, Wnt induction was achieved by treating the cells with LiCl solution at different concentrations for 6 h (for Real time quantitative PCRs) or 24 h (for luciferase reporter assays).

### 2.3. Cell Growth Curves

Cells were plated into 96 well E-plates (ACEA Biosciences, San Diego, CA, USA) and grown in a xCELLigence-RCTA SP system (ACEA Biosciences, San Diego, CA, USA). Cells were seeded in quadruplicates for all conditions. In addition, wells with only media were used as a control. The measurement was taken every 10 min for 120 h. We fitted the growth curve using a logistic function with three characteristic parameters: the length of lag phase, the generation time and the maximum cell index.

### 2.4. Quantification of P1 and P2 Promoter Activities

To quantify the P1 and P2 promoter activities, we developed a PCR assay with primers immediately downstream of the P1 promoter (5′-CTTGGCGGGAAAAAGAACGG-3′ and 5′-AGTTAGATAAAGCCCCGAAAACC-3′) and the P2 promoter (5′-AGCGAATAGGGGGCTTCGC-3′ and 5′-TCGTGGATGCGGCAAGGGTT-3′). In addition, we used a 264 bp DNA sequence that could be amplified by both sets of primers to determine the amplification efficiency and the relative transcription from the P1 and the P1 + P2 promoter, and sub sequentially, the transcription from the P2 promoter.

### 2.5. Reporter Luciferase Assay

Luciferase reporter constructs were generated using pGL3-Basic luciferase reporter vector. A 150 bp stretch of the *MYC* promoters P1 (Chr8,128748216:128748365) or P2 (Chr8,128748378:128748527) was cloned between XhoI and HindIII sites to generate the promoter alone constructs. Furthermore, to test the effect of the Wnt pathway of the fold activation, a Wnt responsive enhancer consisting of 8 TCF7L2 [43] or a control sequence with mutated TCF7L2 DNA binding sites was cloned right upstream from both promoters. Between KpnI and NheI sites, the maps for the plasmids were shared in the Annex S1. The QuickChange II mutagenesis kit (Stratagene, La Jolla, CA, USA) was used to generate the promoter motif mutants. The primers used for the site direct mutagenesis are listed in Table S1. The final mutant promoter sequences were shared in the Annex S2. Reporter constructs were transiently transfected into cells along with a control luciferase plasmid (Renilla luciferase SV40), before luciferase activity was measured using a Dual-Glo luciferase kit (Promega, Madison, WI, USA) in a GloMax-Multi Detection System (Promega, Madison, WI, USA).

### 2.6. Real Time qPCR

Total RNA was isolated from HCT-116 cells and other cell lines using RNeasy Kit (QIAGEN, Hilden, Germany), complementary DNA (cDNA) was generated using Maxima First Strand cDNA Synthesis Kit (Thermo Fisher, Waltham, MA, USA) and qPCRs reactions were prepared using SYBR FAST qPCR Mix (KAPA Biosciences, Wilmington, MA, USA) and ran in a CFX96 Real-Time PCR Detection System (BioRad, Hercules, CA, USA). In all cases, manufacturer guidelines were followed to optimize the amplification conditions. Primer sequences used for Real time quantitative PCRs were designed with Primer3 online tool (http://bioinfo.ut.ee/primer3-0.4.0/) [44] and are listed in Table S2. Quantitation cycle (Cq) values were used to further determine the levels of the specific messenger RNA (mRNA) assayed.

### 2.7. Genome Editing

To generate enhancer deleted HCT-116 clones, guide RNAs (gRNAs) were designed using the online CRISPR design tool (http://crispr.mit.edu/) [45] and cloned into a modified px330-U6-Chimeric_BB-CBh-hSpCas9 vector (Addgene, Cambridge, MA, USA) harboring a Venus fluorescent protein cassette. For each enhancer deletion two vectors coding for gRNAs surrounding the enhancer locus were transfected into HCT-116 cells. A day after transfection, GFP positive cells were sorted by fluorescence-activated cell sorting (FACS) and single plated into 96 well plates. 10 days after plating, the colonies were genotyped by genomic PCR to find clones with the desired deletions. The primers listed in Table S3 were used to generate the different px330 vectors used in this study.

## 3. Results

### 3.1. MYC Alternative Promoters Can Be Differentially Regulated

**Figure 1:**
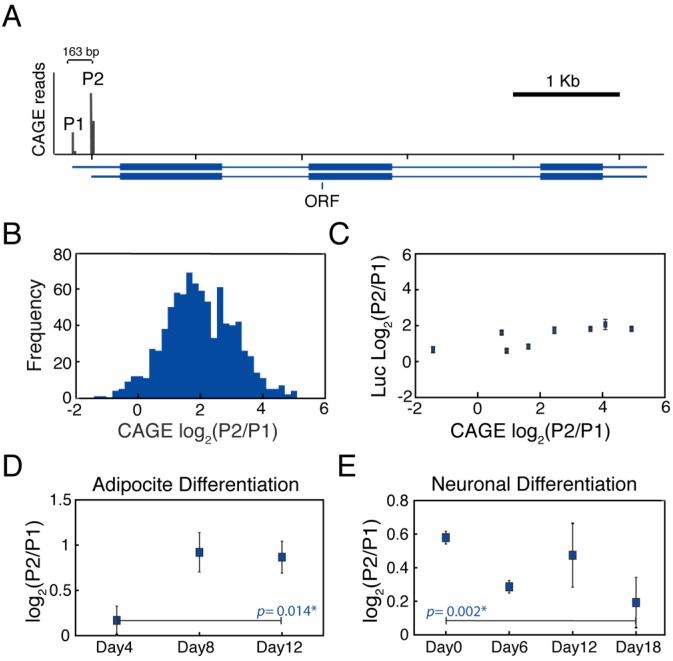
*MYC* transcription is driven by two alternative promoters which are differentially regulated in vivo. (**A**) Cap analysis of gene expression (CAGE) reads show that *MYC* gene is expressed by two alternative promoters, P1 and P2; (**B**) Histogram of promoter usage ratio across 869 samples obtained from the FANTOM5 database; (**C**) In vivo promoter usage ratio cannot be explained by promoter activity alone; (**D**) Adipocyte differentiation leads to stronger usage of the P2 promoter; (**E**) Neuronal differentiation leads to stronger usage of the P1 promoter. ORF: open reading frames. Error bars represent standard error of mean.

To gain a better understanding of how the two alternative *MYC* promoters might be differentially deployed, we surveyed P1 and P2 promoter usage across 869 human cell types and tissues from the FANTOM database [15] (Figure 1A). We counted the number of CAGE reads coming from each promoter within a 11 bp window to calculate the P2 to P1 promoter usage ratio and its distribution across cell lines (Figure 1B). We observed that the P2 promoter preferentially drives transcription of the *MYC* gene across the different cell types in agreement with previous reports [32,38]. Importantly, this analysis showed that the P2 to P1 promoter usage ratio distribution across the cell lines was very broad, spanning over two orders of magnitude (P2/P1_CAGE_ = (0.37 to 34.24)). This suggested that multiple regulatory sequences, including cell specific enhancers and proximal promoter elements, may be important to differentially regulate the activity of the tandem *MYC* promoters.

Based on these preliminary observations, we investigated whether the in vivo promoter usage could be explained by differential promoter activation as a consequence of enhancer bias, derived from the transcriptional network. For this, we measured the independent activity of the P1 and P2 promoters by determining the activity of luciferase constructs harboring the core P2 or P1 promoter sequences across eight cell lines with different P2/P1_CAGE_ ratios. This allowed us to calculate the synthetic P2 to P1 promoter activity ratio across multiple cell lines relative to endogenous promoter usage in the context of intact chromosomes (Figure 1C). These results showed that in all of the cell lines that were tested, the P2 promoter displayed a higher basal activity (P2/P1_Luiferase_ > 1) and this ratio was maintained across different cell lines (P2/P1_Luciferase_ = (1.8, 4.4)), especially when compared to the endogenous CAGE values. This result suggests that regulation of the two *MYC* promoters outside of their genomic context does not recapitulate the broad range of P2 to P1 promoter usage ratio observed for endogenous *MYC* in their native chromatin context. This finding suggests that distal *cis*-regulatory elements are likely to be, at least in part, responsible for differential activation of *MYC* promoter P2 versus P1. 

Next, we explored if changes in the transcriptional regulatory network during differentiation could lead to changes in alternative promoter usage. We analyzed 21 time courses of human cells exposed to differentiation cues and stimuli from the FANTOM database [42]. In 7 out of these 21 time courses, we observed a significant change in the P1/P2 promoter usage along the time course, suggesting that changes in the transcription regulatory network influenced differential promoter usage in a cell-type dependent manner. In addition, we observed that upregulation of either the P1 or P2 promoter usage was possible. For example, during Adipocyte differentiation, P2 promoter usage was stimulated (Figure 1D), while during neuronal differentiation the P1 promoter activity was increased (Figure 1E). Importantly, upregulation of either P1 and P2 promoter usage was independent of *MYC* expression, since the total levels of *MYC* decreased in both time courses over time (Supplementary Materials Figure S1). This analysis suggests that transcription regulatory networks and distal enhancers could be responsible for differentially modulating the activation of the two alternate *MYC* promoters.

### 3.2. Wnt-Responsive Enhancers Preferentially Activate the P1 Promoter

**Figure 2:**
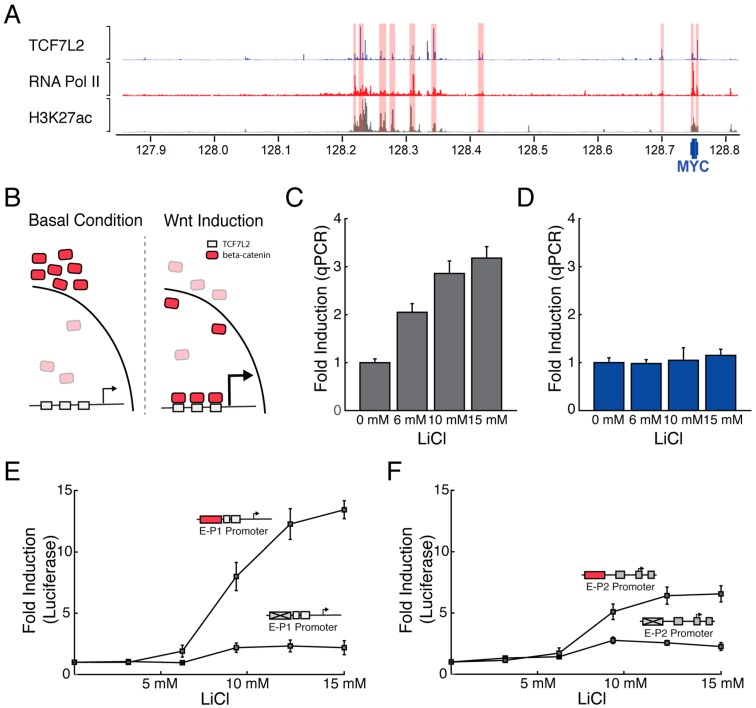
In HCT-116 cells, activation of Wnt-responsive enhancers preferentially upregulates the P1 promoter. (**A**) Multiple Wnt-responsive enhancers (shaded in red) regulate *MYC* transcription. chromatin immunoprecipitation sequencing (ChIP-Seq) traces of TCF7L2, RNA Pol II and H3K27ac mark the location of Wnt-responsive enhancers; (**B**) Wnt induction promotes relocalization of β-catenin; (**C**) Wnt induction strongly upregulates initiation from the P1 promoter; (**D**) Wnt induction does not upregulate initiation from the P2 promoter; (**E**) Induction of Wnt-responsive enhancer strongly activates transcription of the P1 promoter (**F**) Induction of Wnt-responsive enhancer mildly activates transcription of the P1 promoter. qPCR: Real time quantitative PCR. Error bars represent standard error of mean.

To investigate if distal regulatory elements could specifically regulate the differential usage of *MYC* promoters, we tested how enhancers could activate the transcription from the P1 and P2 promoters in the HCT-116 cells. We chose to work with the HCT-116 colon carcinoma cells where multiple Wnt-responsive enhancers have been reported to regulate *MYC* expression [34]. These Wnt-responsive enhancers are located throughout the *MYC* locus, as can be seen based on their TCF7L2, RNA Polymerase II, and H3K27Ac chromatin immunoprecipitation sequencing (ChIP-Seq) profiles (Figure 2A). To test how the Wnt-responsive enhancers regulate the activity of the two alternative *MYC* promoters, we induced the Wnt pathway by treating the cells with different concentrations of LiCl [46], which promotes the relocalization of β-catenin and the subsequent activation of Wnt target genes (Figure 2B). We observed that Wnt-activation increased the overall levels of *MYC* mRNA in a LiCl dependent manner in HCT-116 cells (Supplementary Materials Figure S2). Importantly, the described minor *MYC* promoter P0 didn’t drive *MYC* expression in this cell line, no endogenous transcription was detected and the promoter showed extremely low activity by luciferase assays (Supplementary Materials Figure S3). More interestingly, we found that transcription from the P1 promoter is preferentially activated (Figure 2C), whereas transcription from P2 was not significantly increased (Figure 2D). This result suggests that Wnt induction preferentially activates transcription from the P1 promoter in HCT-116 cells. 

To further probe how Wnt-responsive enhancers may regulate the *MYC* promoters, we developed a system to independently quantify the fold activation of the P1 and P2 promoters by the presence or absence of the Wnt-responsive enhancer. We used the previously generated core P1 and P2 promoters to clone a Wnt-responsive enhancer or control sequence. The Wnt-responsive enhancer consists of 8 strong TCF7L2 binding sites, whereas the control sequence has the 8 TCF7L2 binding sites mutated making it unresponsive to Wnt [43]. We observed that the P1 promoter harboring the Wnt-responsive enhancer was specifically and strongly activated over 12 fold in comparison to the control sequence after activation of the Wnt pathway (Figure 2E). By contrast, the P2 promoter harboring the Wnt-responsive enhancer was mildly activated by the Wnt pathway, increasing the P2 activity by 6 fold (Figure 2F). We believe that the activation of the P2 promoter observed by reporter assays in contrast to the endogenous locus could be in part due to the transcriptional saturation of the P2 promoter on its endogenous context, resulting from the action of multiple regulatory pathways [32]. Taken together, these results further support the finding that Wnt-responsive enhancers preferentially activate transcription from the P1 promoter.

### 3.3. Enhancer Deletions Preferentially Downregulate Transcription from the P1 Promoter

**Figure 3:**
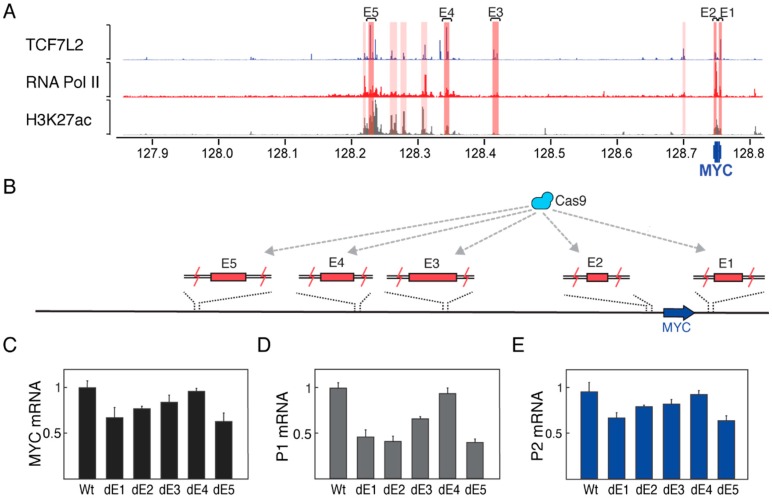
Wnt-responsive enhancer deletions preferentially downregulate transcription from the P1 promoter. (**A**) Five enhancers were selected across the *MYC* locus to be deleted by using CRISPR/Cas9; (**B**) Effect of enhancer deletion on *MYC* mRNA levels; (**C**) Enhancer deletions strongly downregulate transcription from the P1 promoter; (**D**) While in the P2 promoter the enhancer deletions only cause minor downregulation. Error bars represent standard error of mean.

Next, we investigated the effect of deleting Wnt-responsive enhancers on the two alternative *MYC* promoters. Five enhancers were selected across the topological domain located at approximately 1 Kb, 7 Kb, 335 Kb, 405 Kb and 550 Kb from the *MYC* gene body based on their strong binding profiles for TCF7L2 and RNA Pol II. Some of these enhancers have been previously shown to form with the *MYC* gene by capturing Hi-C and regulating promoter activity by luciferase assays [46,47,48,49,50]. These enhancers start from most 3′ distal (Figure 3A). Next, we used CRISPR/Cas9 to generate five different cell lines with homozygous enhancer deletions (Figure 3B). Here, sequence-specific guide RNAs were designed to flank each enhancer to generate homozygous enhancer deletions surrounding the TCF7L2 binding sites, generating deletion between 775 bp and 2403 bp (Supplementary Materials Figure S4). When we tested the effect of these enhancer deletions on *MYC* mRNA, we found that in four cases, the deletions caused significant downregulation of total *MYC* mRNA levels (Figure 3C). Downregulation of *MYC* mRNA levels caused by the single enhancer deletions was accompanied by defects in cell growth (Supplementary Materials Figure S5), suggesting that these enhancers are important for *MYC* regulation across the cell cycle. When the specific activity of the two alternative *MYC* promoters was measured, we observed that the enhancer deletions preferentially downregulated transcription initiation from the P1 promoter (Figure 3D), while only a minor reduction of transcription initiation was observed from the P2 promoter (Figure 3E). In sum, these genome editing experiments further support the preferential regulatory relationship between the select set of Wnt-responsive enhancers and the P1 promoter of *MYC*.

### 3.4. Distinct Core Promoter Architecture Influences Specific Enhancer-Promoter Communication

**Figure 4:**
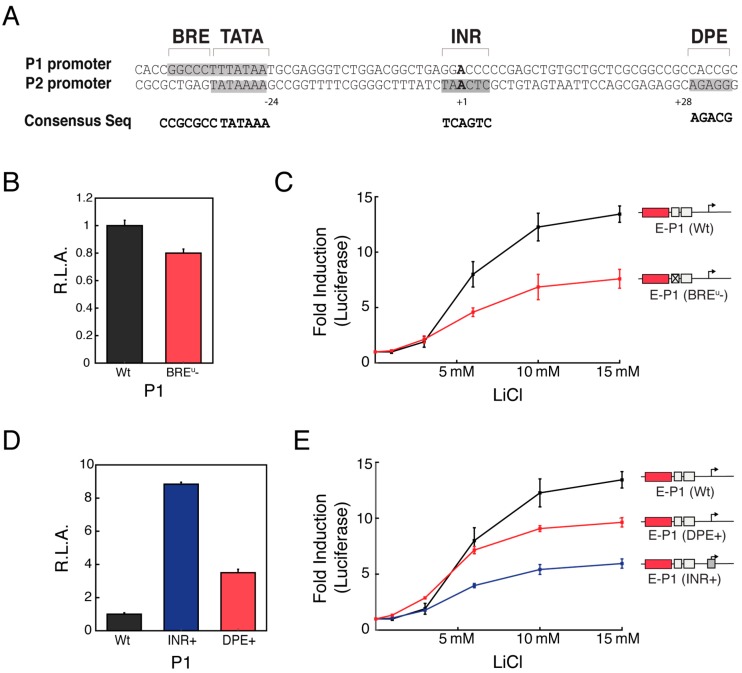
Promoter architecture mediates differential enhancer communication. (**A**) *MYC* Promoter possesses dissimilar promoter architecture. While the P1 promoter has a TATA box and a BRE^UP^ motif, the P2 promoter possess a strong TATA box, an initiator sequence (INR) and a downstream core promoter elements (DPE) motif; (**B**) P1 promoter lacking the BRE^u^ motif decreased minimally the basal activity; (**C**) P1 promoter lacking the BRE^u^ motif had a drastic effect on the maximum fold activation; (**D**) P1 promoter with either a consensus INR or DPE motif have stronger basal activity; (**E**) The addition of a consensus INR or DPE motif to the P1 promoter diminishes fold activation. R.L.A.: Relative luciferase activity. Error bars represent standard error of mean.

Previous studies have shown that enhancer-promoter specificity can be mediated by the presence or absence of different core promoter elements [7,9,10]. To determine whether core promoter architecture comprised of specific elements might also mediate the selectivity of Wnt-responsive enhancers, we analyzed the promoter architecture of the two *MYC* core promoters. Interestingly, we observed that the two alternative *MYC* core promoter possess rather distinct core promoter element composition and disposition (Figure 4A). Importantly, the type and disposition of promoter elements that are present in the two distinct *MYC* promoters are known to be highly conserved in mammals [18] (Supplementary Materials Figure S6), suggesting that the arrangement of elements in these two dissimilar promoter architectures could be important for specific regulation.

To determine the basis for the preferential activation of P1 upon Wnt-induction, we investigated the role of the different core promoter elements that are present within the two *MYC* promoters. We first tested the role of the BRE^u^ on Wnt-dependent activation, which is only present within the P1 promoter. We observed that mutation of the BRE^u^ motif in P1 had little effect on its basal promoter activity (Figure 4B). In contrast, the presence of the BRE^u^ motif on the P1 promoter was crucial for Wnt-responsive enhancer-mediated activation (Figure 4C). Furthermore, we observed that insertion of a consensus BRE^u^ motif into P2 had little effect on its basal activity, but increased its maximum fold activation (Supplementary Materials Figure S7). The function gain associated with the presence of the BRE^u^ motif suggests that this is a promoter element that potentiates the capacity of P1 to be selectively activated by Wnt-responsive enhancers.

Lastly, we tested the role of INR and DPE motif, only present in the P2 promoter, may have on the promoter capacity to be activated by the Wnt-responsive enhancers. We generated P1 promoter containing either a consensus INR or DPE motif. The basal activity of these promoters was increased 8-fold and 2-fold for the INR and DPE P1 promoter variants, respectively (Figure 4D). Intriguingly, the presence of these two consensus motifs decreased the capacity of the P1 promoter to be activated by Wnt-responsive enhancers (Figure 4E) but did not decrease the maximum promoter activity (Supplementary Materials Figure S8), pointing out that introduction of some promoter elements may have complex effects on promoter basal activity and fold activation. Conversely, disrupting the strong INR or DPE sequences of the native P2 promoter resulted in a decrease in its basal promoter activity accompanied by an increased fold activation induced by the Wnt-enhancers (Supplementary Materials Figure S7). These results suggest that the presence of INR and DPE, at least for the P2 promoter, might dampen the fold activation of enhancer-induced transcription by elevating the basal promoter activity. Overall, these results indicate that selective activation of promoters by enhancers can be genetically encoded by the presence of different promoter elements.

## 4. Discussion

The usage of alternative promoters as drivers of transcription initiation is a prevalent phenomenon that occurs throughout metazoans [12,48]. Although numerous reports have shown that differential regulation of alternative promoter is important for proper development and cellular maintenance [12,15,17], the mechanisms that regulate promoter usage are limited and have focused on the role of promoter proximal regulation [23,38,39]. Here, we show that selective enhancers can differentially regulate the activity of the two *MYC* core promoters, further expanding upon the early reports which have focused on *MYC* proximal promoter elements that regulated transcription factor binding or mRNA stability [25,37,38,51]. Specifically, we found that the *MYC* promoters, P1 and P2, are differentially deployed across different tissues and cell types, and those distal regulatory elements are necessary for selective regulation of alternative core promoters, in expanding the repertoire of possible mechanisms that could regulate *MYC* transcription [32]. In addition, by working with HCT-116 colon carcinoma cells, we demonstrated that Wnt-responsive enhancers preferentially activate the P1 promoter in the genomic context and by reporter assays. Finally, we showed that the preferential activation of the P1 promoter is dependent on specific promoter elements and its promoter architecture revealing and important aspect of the mechanism directing alternative core promoter specificity.

Previous studies have reported that certain promoter elements present within the core promoter architecture can be important to specify enhancer-promoter communication [9,10,52]. Here, we have extended these earlier studies by establishing that promoter elements present in the P1 promoter allow a more precise communication with distal enhancers. Moreover, this selective promoter-enhancer communication is mediated by the combined action of multiple core promoter elements via two mechanisms. First, the presence of a BRE^u^ motif potentiates activation of P1 independent of its basal activity. And second, the presence of INR or DPE motifs apparently limits the range of enhancer dependent promoter activation by increasing the basal activity of the core promoter. This means that in HCT-116 cells, the P2 promoter serves as a basal promoter, largely insensitive to Wnt-responsive enhancers, whereas the P1 promoter can be fine-tuned by Wnt-responsive enhancers. The presence of these two differentially regulated promoters could be important for precise regulation of transcription initiation, especially for the genes that are tightly regulated, such as *MYC*.

Multiple mechanisms are thought to regulate the specific formation of enhancer-promoter interactions that include chromosome topology, changes in the epigenetic chromatin landscape and biochemical compatibility [52,53,54,55,56,57]. The observation that small changes in alternative core promoter elements can differentially communicate with distal enhancers could further diversify the combinatorial specificity of these highly regulated interactions [53]. Interestingly, genome-wide analysis of mammalian promoters has shown that the presence of alternative promoters is overrepresented in highly regulated genes [58]. The *MYC* gene is exquisitely regulated at the transcriptional level, with more than 270 enhancers annotated in the enhancer atlas to regulate *MYC*, of which, dozens have been functionally validated [33,35,36,59]. The differential usage of two alternative *MYC* core promoters would significantly expand the network and specificity of possible long distance enhancer/promoter interactions that are necessary to mediate different temporal and spatial transcriptional outcomes.

## Supplementary Materials

The following are available online at http://www.mdpi.com/2073-4425/9/6/270/s1. Figure S1: Effect of cell differentiation on total *MYC* expression, Figure S2: Effect of LiCl mediate Wnt activation on *MYC* mRNA in HCT-116 cells, Figure S3: Activity of *MYC’s* promoters by luciferase assay, Figure S4: Enhancer deletions information and results of Sanger Sequencing, Figure S5: Growth curves of single enhancer deleted clonal lines, Figure S6: Conservation of core promoter elements in *MYC* promoters, Figure S7: Effect of core promoter mutants on P2 basal activity and fold activation, Figure S8: Influence of INR and DPE on P1 promoter maximum promoter activity, Table S1: Oligonucleotides used to generate promoter reporter assays and mutants, Table S2: Primers for RT-PCR, Table S3: Primers used to generate px330 plasmids, Annex S1: *MYC* reporter plasmids, Annex S2: Sequences of the promoter mutants.
