# Supplementary material for: Selective Activation of Alternative MYC Core Promoters by Wnt-Responsive Enhancers
Source: Genes (Basel). 2018 May 23;9(6):270. doi: 10.3390/genes9060270 (PMC6027352; doi:10.3390/genes9060270)
Supplement: Supplementary file 1 [file genes-09-00270-s001.pdf]

Supplemental Information:

"Selective activation of alternative *MYC* core promoters by Wnt-responsive enhancers"

Figure S1.- Effect of cell differentiation on total *MYC* expression.
Figure S2.- Effect of LiCl mediate Wnt activation on *MYC* mRNA in HCT-116 cells.
Figure S3.- Activity of *MYC's* promoters by luciferase assay
Figure S4.- Enhancer deletions information and results of Sanger Sequencing
Figure S5.- Growth curves of single enhancer deleted clonal lines.
Figure S6.- Conservation of core promoter elements in *MYC* promoters
Figure S7.- Effect of core promoter mutants on P2 basal activity and fold activation
Figure S8.- Influence of INR and DPE on P1 promoter maximum promoter activity.

Table S1 Oligonucleotides used to generate promoter reporter assays and mutants
Table S2 Primers for RT-PCR
Table S3 Primers used to generate px330 plasmids

Annex S1 MYC reporter plasmids
Annex S2 Sequences of the promoter mutants

Supplementary References.

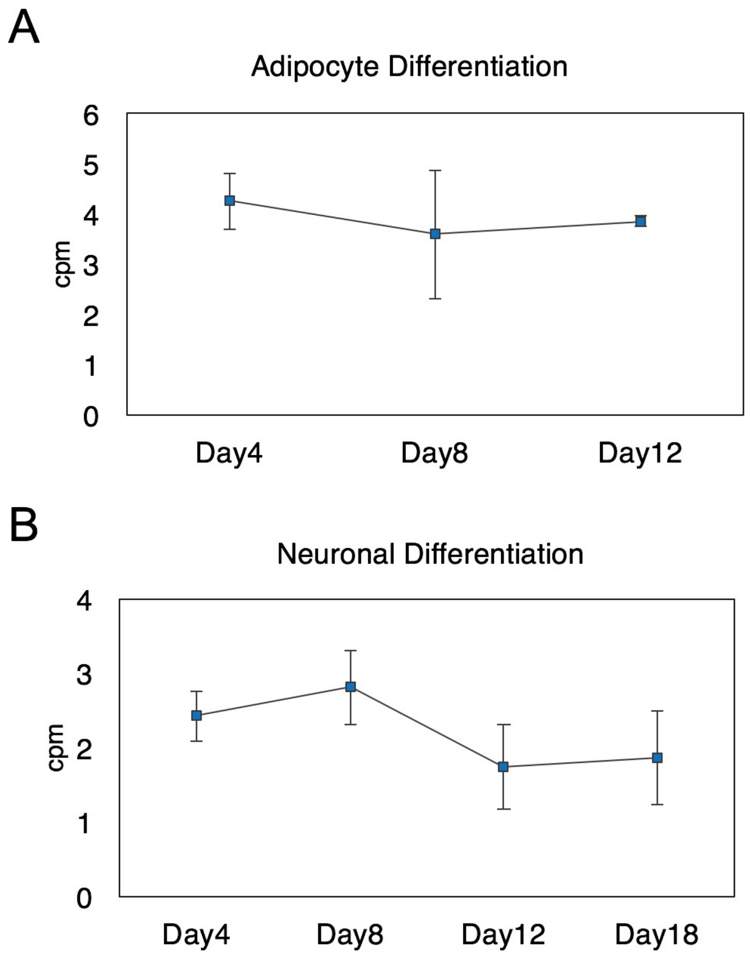

Figure S1.- Effect of cell differentiation on total *MYC* expression.
(A) Adipocyte differentiation leads to minor downregulation of *MYC* expression.
(B) Neuronal differentiation leads to downregulation of *MYC* expression.

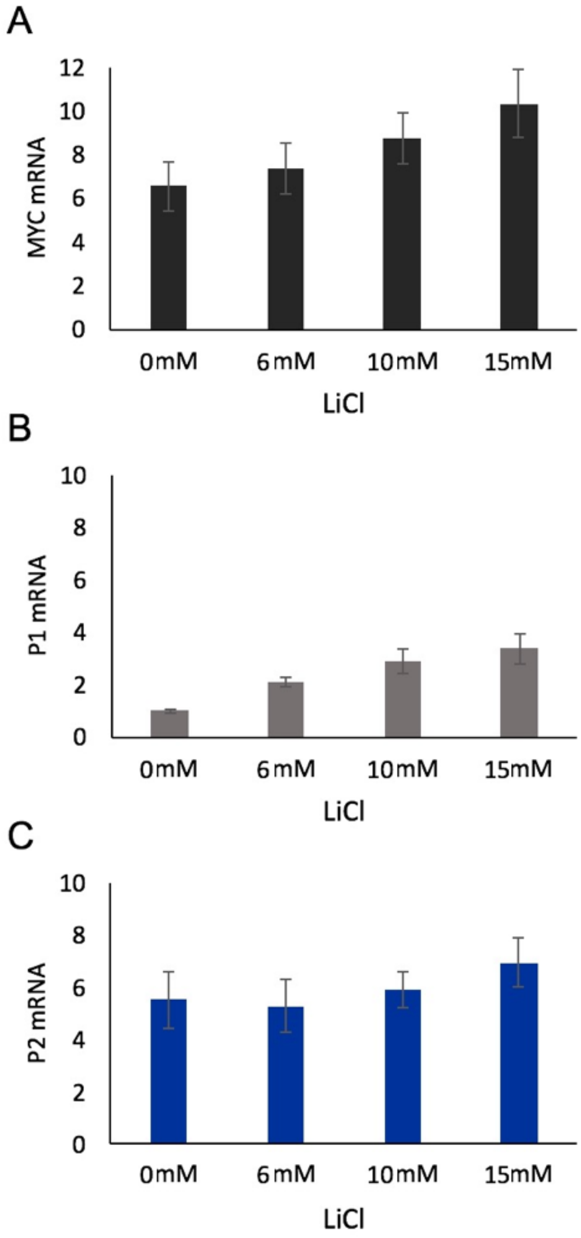

Figure S2.- Effect of Wnt activation on *MYC* mRNA in HCT-116 cells.
(A) Wnt induction upregulates total *MYC* transcription.
(B) Wnt induction upregulates transcriptional activity of the P1 promoter.
(C) Wnt induction does not upregulates transcriptional activity of the P2 promoter.

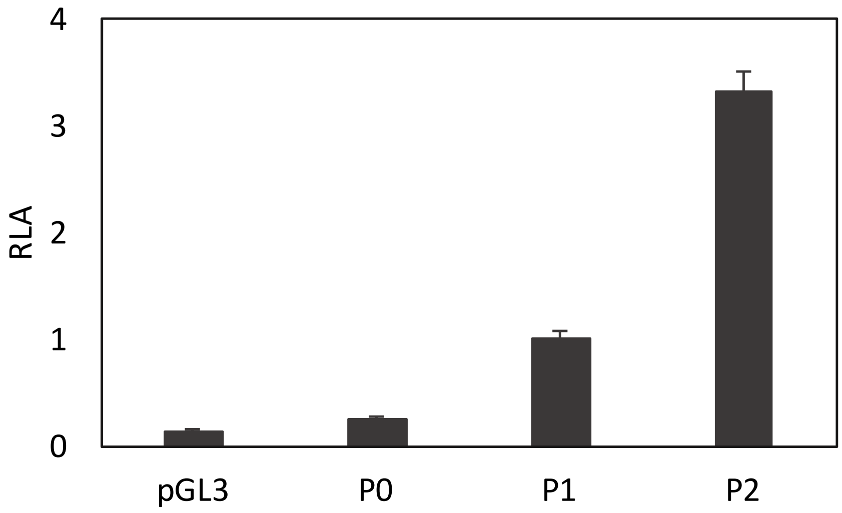

Figure S3.- Activity of *MYC's* promoters by luciferase assay.
The P0, P1 & P2 MYC promoters were cloned from the -100 to +50 with respect to the transcription start site into a pGL3 vector to assess promoter strength. As can be seen, transcription initiating from P0 is minimal in comparison to P1 or P2.

Enhancer Deletion Information	Sanger sequencing chromatogram	Ref.
Enhancer 1<br>Target Sequence:<br>Left: CTCATCCTGAGTCCTTGAAA<br>Right:<br>TAATCAAGAATCGGACGTGA<br>Genomic location:<br>Left: 128754988 ^ Right: 128755777<br>Deletion size: 808bp	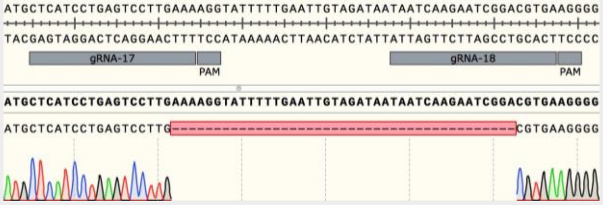	(1,2)
Enhancer 2<br>Target Sequence:<br>Left: TGAACTAGGAAATTAATGCC<br>Right: CTGTGAGTATAAATCATCGC<br>Genomic location:<br>Left: 128746967 ^ Right: 128747978<br>Deletion size: 1016bp	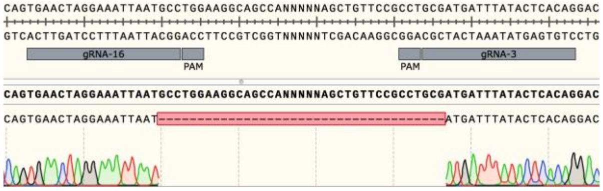	(2–4)
Enhancer 3<br>Target Sequence:<br>Left: GCAATTCCGAGGTGATCAGG<br>Right: ATATCCCCGGTTCATAGATA<br>Genomic location:<br>Left: 128412657 ^ Right: 128415055<br>Deletion size: 2403bp	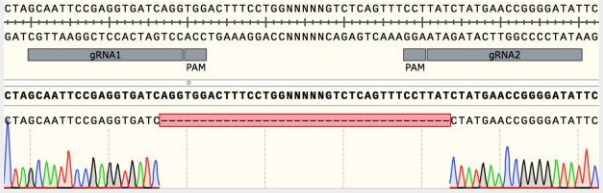	(5–7)
Enhancer 4<br>Target Sequence:<br>Left: GTGGACGGTGCTACAGACTC<br>Right: GAGAATCCATGATTACTGCT<br>Genomic location:<br>Left: 128342323 ^ Right: 128343112<br>Deletion size: 775bp	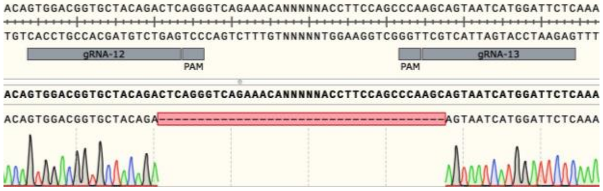	(7)
Enhancer 5<br>Target Sequence:<br>Left: AGGTGCATAACCCTTTAAAC<br>Right: GATCTCATTAATTGACTGCG<br>Genomic location:<br>Left: 128227129 ^ Right: 128228107<br>Deletion size: 983bp	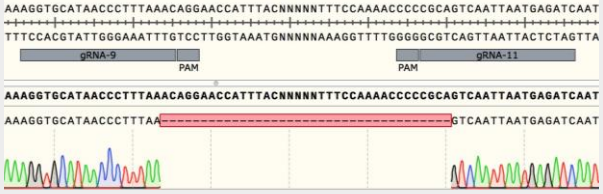	(7–9)

Figure S4.- Enhancer deletions information and results of Sanger Sequencing.
This figure provides information about the sequences that were targeted by CRISPR/Cas9, the genomic coordinates, the respective sizes of the enhancer deletions, sample sanger sequencing results and relevant references.

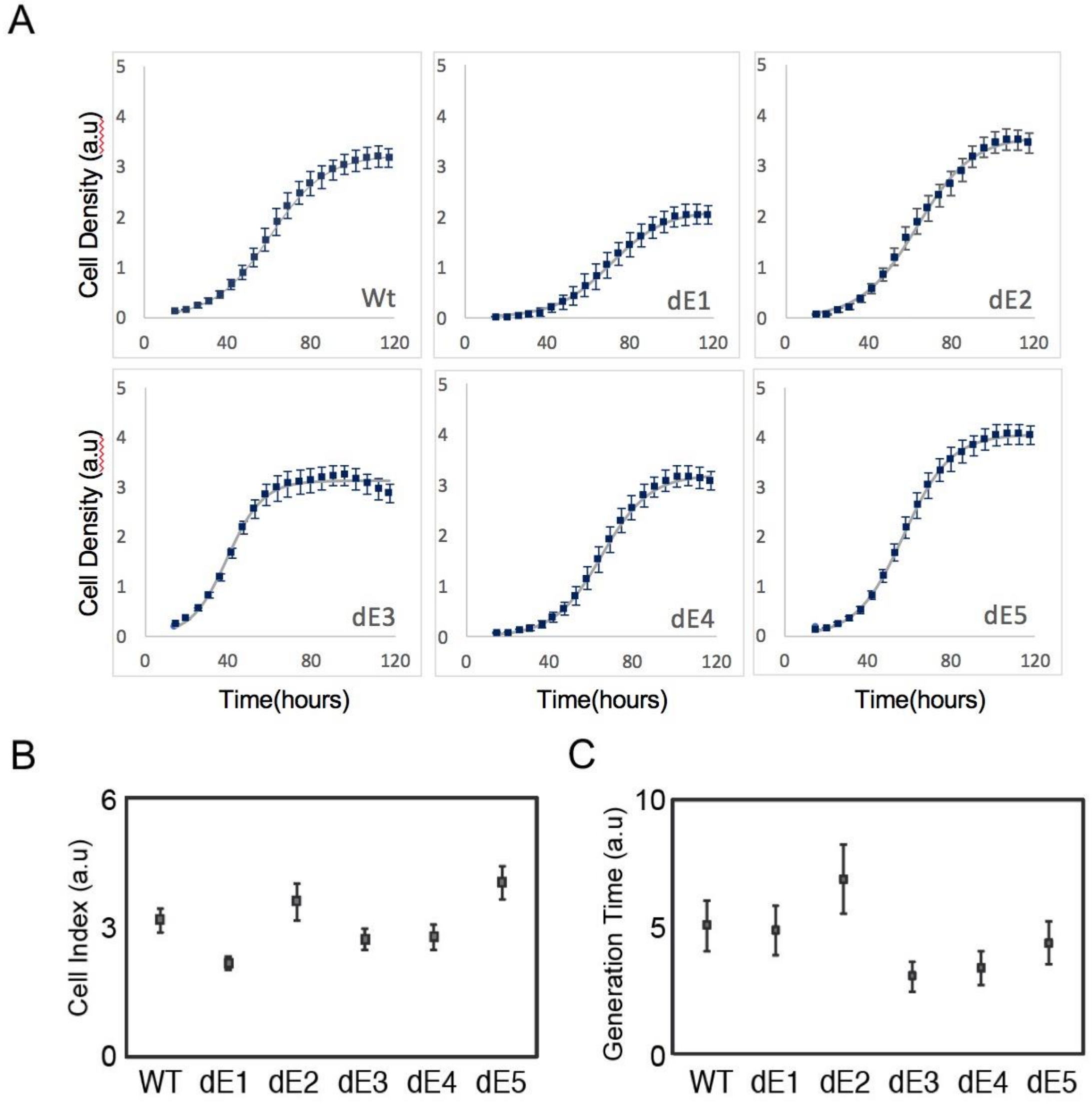

Figure S5.- Growth curves of single enhancer deleted clonal lines.
(A) Growth curves were performed in wild type HCT-116 cells as well as the five Wnt-responsive enhancer deleted single clonal lines.
(B) The cell index, a measurement of maximum cell density, was calculated. As can be observed, the cell index is significantly modified for some enhancer deletions.
(C) The generation time, a measurement of speed of cell division, was calculated. As can be observed, the generation time is significantly modified for some enhancer deletions.

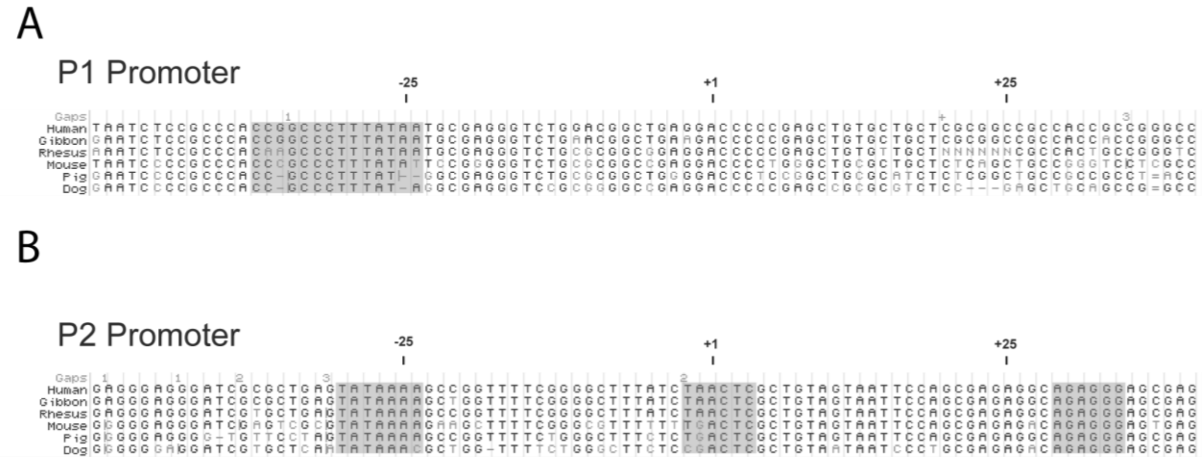

Figure S6.- Conservation of core promoter elements in *MYC* promoters.
(A) Comparison of P1 promoter sequences across five different mammals with ~90 million years of evolutionary divergence. As can be seen, the TATA box and the BRE motifs are conserved.
(B) Comparison of P2 promoter sequences across five mammals with ~90 million years of evolutionary divergence. As can be seen, the TATA box, the INR and DPE motifs are highly conserved.

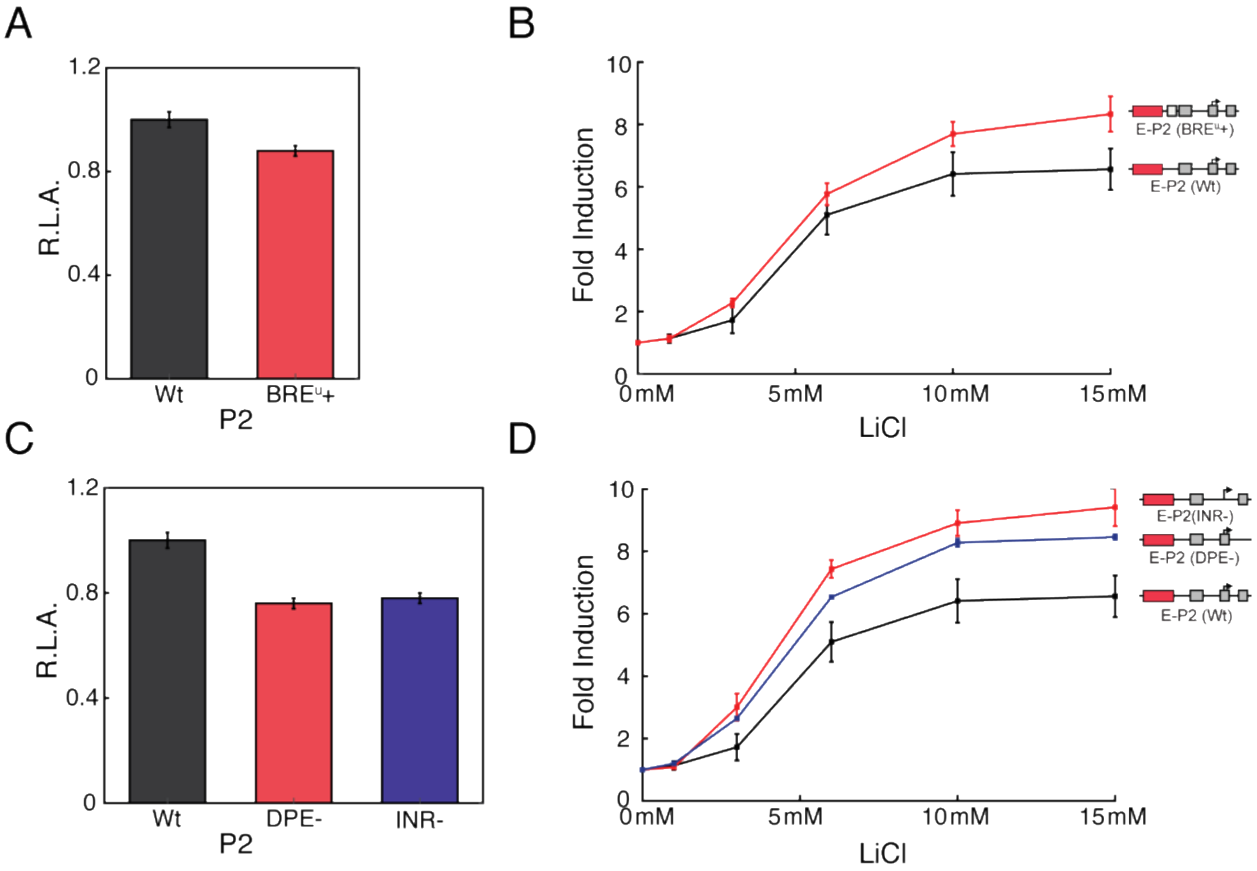

Figure S7.- Effect of core promoter mutants on P2 basal activity and fold activation.
(A) P2 promoter with a BRE^{u} motif generates a slight decrease of the basal promoter activity.
(B) P2 promoter with a BRE^{u} motif has a decreased fold activation.
(C) P2 promoters lacking DPE or INR motif shown downregulation in the promoter basal activity.
(D) P2 promoter lacking DPE or INR motif have a decreased fold activation.

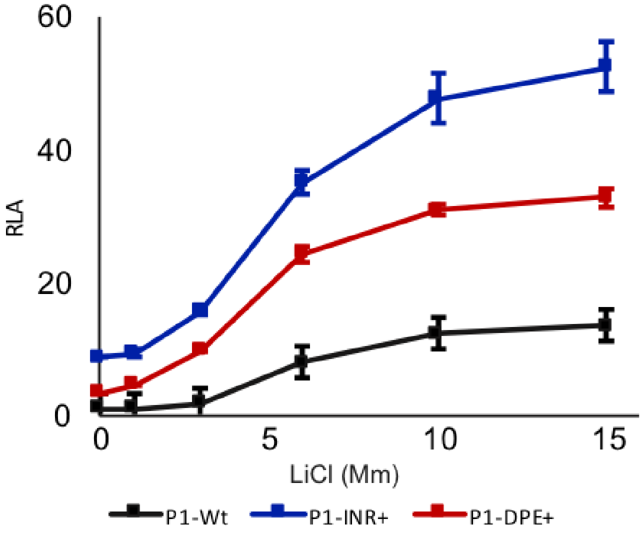

Figure S8.- Influence of INR and DPE on P1 promoter maximum promoter activity.
Non normalized curves of LiCl activation on the P1 promoter mutants harboring INR or DPE motifs. As can be observed, under no LiCl induction the basal transcription levels for each of the promoter is drastically different.

Table S1 Oligonucleotides used to generate promoter reporter assays and mutants

Cloning P1 and P2 promoters	
atgcGGTCTCATCGACCGGGTTCCCAAAGCAGAGG	Cloning Myc Promoter into pGL3
atgcAAGCTTGGAGCCAGGGACGGCCGG	Cloning Myc Promoter into pGL3
atgcGGTCTCATCGACTGCCTCGAGAAGGGCAGGG	Cloning Myc Promoter into pGL3
atgcAAGCTTGCTCTTCCACCCTAGCCG	Cloning Myc Promoter into pGL3

Luciferase Mutations P1 promoter	
ccagaccctcgcattataaacgtgttgtgggcggagattagcgag	Mut BRE-P1
ctcgctaatctccgcccacaacacgtttataatgcgagggtctgg	Mut BRE-P1
ctcagccgtccagaccctcgcaggggcgcgggccggtgggcggagattagc	Mut TATA-P1
gctaatctccgcccaccggcccgcgcccctgcgagggtctggacggctgag	Mut TATA-P1
cagcacagctcggaactgatcagccgtccagaccctcgcattat	Mut INR-P1
ataatgcgagggtctggacggctgatcagttccgagctgtgctg	Mut INR-P1
cggccggggcccgaggtctgcggccgcgagca	Mut DPE-P1
tgctcgcggccgcagacctcgggccccggccg	Mut DPE-P1

Luciferase Mutations P2 promoter	
ccgaaaaccggcttttataggcgccccgatccctccctccgttct	Mut BRE-P2
agaacggagggagggatcggggcgcctataaaagccggttttcgg	Mut BRE-P2
gaacggagggagggatcgcgctgaggcgccccgccggttttcggggctttatctaac	Mut TATA-P2
gttagataaagccccgaaaaccggcggggcgcctcagcgcgatccctccctccgttc	Mut TATA-P2
tcgctggaattactacagcgggtccgataaagccccgaaaaccg	Mut INR-P2
cggttttcggggctttatcggacccgctgtagtaattccagcga	Mut INR-P2
gccggccgcccgctcgctcatgaggcctctcgctggaattac	Mut DPE-P2
gtaattccagcgagaggcctcatgagcgagcgggcggccggc	Mut DPE-P2

Table S2 Primers for RT-PCR

qPCR Primers	
TCCCTGGAGAAGAGCTACGA	qPCR b-actin
AGCACTGTGTTGGCGTACAG	qPCR b-actin
AATCCCATCACCATCTTCCA	qPCR GAPDH
TGGACTCCACGACGTACTCA	qPCR GAPDH
CAGCTGCTTAGACGCTGGATT	qPCR Myc
GTAGAAATACGGCTGCACCGA	qPCR Myc
AGCGAATAGGGGGCTTCGC	qPCR P1+2 Fw
TCGTGGATGCGGCAAGGGTT	qPCR P1+2 Rv
CTTGGCGGGAAAAAGAACGG	qPCR P1 Fw
AGTTAGATAAAGCCCCGAAAACC	qPCR P1 Rv

Table S3 Primers used to generate px330 plasmids

px330 gRNA construction	
caccAGGTGCATAACCCTTTAAAC	px330-E5 Right fw
aaacGTTTAAAGGGTTATGCACCT	px330-E5 Right rv
caccGATCTCATTAATTGACTGCG	px330-E5 Left fw
aaacCGCAGTCAATTAATGAGATC	px330-E5 Left rv
caccGTGGACGGTGCTACAGACTC	px330-E4 Right fw
aaacGAGTCTGTAGCACCGTCCAC	px330-E4 Right rv
caccGAGAATCCATGATTACTGCT	px330-E4 Left fw
aaacAGCAGTAATCATGGATTCTC	px330-E4 Left rv
caccGCAATTCCGAGGTGATCAGG	px330-E3 Right fw
aaacCCTGATCACCTCGGAATTGC	px330-E3 Right rv
caccATATCCCCGGTTCATAGATA	px330-E3 Left fw
aaacTATCTATGAACCGGGGATAT	px330-E3 Left rv
caccAGGCCTTTGCCGCAAACGCG	px330-E2 Right fw
aaacCGCGTTTGCGGCAAAGGCCT	px330-E2 Right rv
caccCTATTCAACCGCATAAGAGA	px330-E2 Left fw
aaacTCTCTTATGCGGTTGAATAG	px330-E2 Left rv
caccCTCATCCTGAGTCCTTGAAA	px330-E1 Right fw
aaacTTTCAAGGACTCAGGATGAG	px330-E1 Right rv
caccTAATCAAGAATCGGACGTGA	px330-E1 Left fw
aaacTCACGTCCGATTCTTGATTA	px330-E1 Left rv

Annex S1: MYC reporter plasmids

All the reporter plasmids were build using the the Pgl3-Basic reporter plasmid. The reporter plasmids sequences presented in the article were inserted in the Pgl3-Basic between KpnI and HindIII sites.

Promoter sequences are underline. enhancer sequences, when present, are double underline.

P1 Promoter:
GGTACCGAGCTCTTACGCGTGCTAGCCCGGGCTCGACCGGGTTCCCAAAGCAGAGGGCGTGGGGGAA
AAGAAAAAAGATCCTCTCTCGCTAATCTCCGCCCACCGGCCCTTTATAATGCGAGGGTCTGGACGGCT
GAGGACCCCCGAGCTGTGCTGCTCGCGGCCGCCACCGCCGGGCCCCGGCCGTCCCTGGCTCCAAGCTT

P2 promoter:
GGTACCGAGCTCTTACGCGTGCTAGCCCGGGCTCGACTGCCTCGAGAAGGGCAGGGCTTCTCAGAGGC
TTGGCGGGAAAAAGAACGGAGGGAGGGATCGCGCTGAGTATAAAAGCCGGTTTTCGGGGCTTTATCT
AACTCGCTGTAGTAATTCCAGCGAGAGGCAGAGGGAGCGAGCGGGCGGCCGGCTAGGGTGGAAGAG
CAAGCTT

Wnt-responsive enhancer + P1 promoter:
GGTACCGAGCTCTTACGCGAGATCAAAGGGGGTAAGATCAAAGGGGGTAAGATCAAAGGGGCGCGA
GATCAAAGGGGGTAAGATCAAAGGGGGTAAGATCAAAGGGGGTAAGATCAAAGGGGCGCGCCCGCG
TGCTAGCCCGGGCTCGACCGGGTTCCCAAAGCAGAGGGCGTGGGGGAAAAGAAAAAAGATCCTCTCT
CGCTAATCTCCGCCCACCGGCCCTTTATAATGCGAGGGTCTGGACGGCTGAGGACCCCCGAGCTGTGC
TGCTCGCGGCCGCCACCGCCGGGCCCCGGCCGTCCCTGGCTCCAAGCTT

Wnt-mutated enhancer + P1 promoter:
GGTACCTTACGCGAGGCCAAAGGGGGTAAGGCCAAAGGGGGTAAGGCCAAAGGGGGTAAGGCCAAA
GGCGCGAGGCCAAAGGGGGTAAGGCCAAAGGGGGTAAGGCCAAAGGGGGTAAGGCCAAAGGCCCG
GGCTCGAGCTAGCCCGGGCTCGACCGGGTTCCCAAAGCAGAGGGCGTGGGGGAAAAGAAAAAAGAT
CCTCTCTCGCTAATCTCCGCCCACCGGCCCTTTATAATGCGAGGGTCTGGACGGCTGAGGACCCCCGA
GCTGTGCTGCTCGCGGCCGCCACCGCCGGGCCCCGGCCGTCCCTGGCTCCAAGCT

Wnt-responsive enhancer + P2 promoter:
GGTACCGAGCTCTTACGCGAGATCAAAGGGGGTAAGATCAAAGGGGGTAAGATCAAAGGGGCGCGA
GATCAAAGGGGGTAAGATCAAAGGGGGTAAGATCAAAGGGGGTAAGATCAAAGGGGCGCGCCCGCG
TGCTAGCCCGGGCTCGACTGCCTCGAGAAGGGCAGGGCTTCTCAGAGGCTTGGCGGGAAAAAGAACG
GAGGGAGGGATCGCGCTGAGTATAAAAGCCGGTTTTCGGGGCTTTATCTAACTCGCTGTAGTAATTCC
AGCGAGAGGCAGAGGGAGCGAGCGGGCGGCCGGCTAGGGTGGAAGAGCAAGCTT

Wnt-mutated enhancer + P2 promoter:

GGTACCTTACGCGAGGCCAAAGGGGGTAAGGCCAAAGGGGGTAAGGCCAAAGGGGGTAAGGCCAAA
GGCGCGAGGCCAAAGGGGGTAAGGCCAAAGGGGGTAAGGCCAAAGGGGGTAAGGCCAAAGGCCCG
GGCTCGAGCTAGCCCGGGCTCGACTGCCTCGAGAAGGGCAGGGCTTCTCAGAGGCTTGGCGGGAAAA
AGAACGGAGGGAGGGATCGCGCTGAGTATAAAAGCCGGTTTTCGGGGCTTTATCTAACTCGCTGTAGT
AATTCCAGCGAGAGGCAGAGGGAGCGAGCGGGCGGCCGGCTAGGGTGGAAGAGCAAGCTT

Annex S2: Sequences of the promoter mutants

- P1 WT
  GGTTCCCAAAGCAGAGGGCGTGGGGGAAAAGAAAAAAGATCCTCTCTCGCTAATCTCCGCCCACC
  GGCCCTTTATAATGCGAGGGTCTGGACGGCTGAGGACCCCCGAGCTGTGCTGCTCGCGGCCGCCAC
  CGCCGGGCCCCGGCCGTCC

- P1 BREm
  GGTTCCCAAAGCAGAGGGCGTGGGGGAAAAGAAAAAAGATCCTCTCTCGCTAATCTCCGCCCAC**aa**
  cacgTTTATAATGCGAGGGTCTGGACGGCTGAGGACCCCCGAGCTGTGCTGCTCGCGGCCGCCACCGC
  CGGGCCCCGGCCGTCC

- P1 TATAm
  GGTTCCCAAAGCAGAGGGCGTGGGGGAAAAGAAAAAAGATCCTCTCTCGCTAATCTCCGCCCACC
  GGCCC**gcgcccc**TGCGAGGGTCTGGACGGCTGAGGACCCCCGAGCTGTGCTGCTCGCGGCCGCCACCG
  CCGGGCCCCGGCCGTCC

- P1 INRm
  GGTTCCCAAAGCAGAGGGCGTGGGGGAAAAGAAAAAAGATCCTCTCTCGCTAATCTCCGCCCACC
  GGCCCTTTATAATGCGAGGGTCTGGACGGCTGA**tcagtt**CCGAGCTGTGCTGCTCGCGGCCGCCACCGC
  CGGGCCCCGGCCGTCC

- P1 DPE
  GGTTCCCAAAGCAGAGGGCGTGGGGGAAAAGAAAAAAGATCCTCTCTCGCTAATCTCCGCCCACC
  GGCCCTTTATAATGCGAGGGTCTGGACGGCTGAGGACCCCCGAGCTGTGCTGCTCGCGGCCGC**agacc**
  tCGGGCCCCGGCCGTCC

- P2 WT
  CTGCCTCGAGAAGGGCAGGGCTTCTCAGAGGCTTGGCGGGAAAAAGAACGGAGGGAGGGATCGCG
  CTGAGTATAAAAGCCGGTTTTCGGGGCTTTATCTAACTCGCTGTAGTAATTCCAGCGAGAGGCAGAG
  GGAGCGAGCGGGCGGCC

- P2 BREm
  CTGCCTCGAGAAGGGCAGGGCTTCTCAGAGGCTTGGCGGGAAAAAGAACGGAGGGAGGGATCG**gg**
  gcgccTATAAAAGCCGGTTTTCGGGGCTTTATCTAACTCGCTGTAGTAATTCCAGCGAGAGGCAGAGG
  GAGCGAGCGGGCGGCCG

- P2 TATAm
  CTGCCTCGAGAAGGGCAGGGCTTCTCAGAGGCTTGGCGGGAAAAAGAACGGAGGGAGGGATCGCG
  CTGAGgcgccccGCCGGTTTTCGGGGCTTTATCTAACTCGCTGTAGTAATTCCAGCGAGAGGCAGAGGG
  AGCGAGCGGGCGGCCG

- P2 INRm
  CTGCCTCGAGAAGGGCAGGGCTTCTCAGAGGCTTGGCGGGAAAAAGAACGGAGGGAGGGATCGCG
  CTGAGTATAAAAGCCGGTTTTCGGGGCTTTATC**ggacc**CGCTGTAGTAATTCCAGCGAGAGGCAGAGG
  GAGCGAGCGGGCGGCCG

- P2 DPE
  CTGCCTCGAGAAGGGCAGGGCTTCTCAGAGGCTTGGCGGGAAAAAGAACGGAGGGAGGGATCGCG
  CTGAGTATAAAAGCCGGTTTTCGGGGCTTTATCTAACTCGCTGTAGTAATTCCAGCGAGAGGC**ctcat**G
  AGCGAGCGGGCGGCCG

Supplementary References:

1. Sierra J, Yoshida T, Joazeiro CA, Jones KA. The APC tumor suppressor counteracts β-catenin activation and H3K4 methylation at Wnt target genes. Genes Dev. 2006 Mar 1;20(5):586–600.

2. Yochum GS, Sherrick CM, MacPartlin M, Goodman RH. A β-catenin/TCF-coordinated chromatin loop at MYC integrates 5′ and 3′ Wnt responsive enhancers. Proc Natl Acad Sci. 2010 Jan 5;107(1):145–50.

3. Yochum GS, Cleland R, Goodman RH. A Genome-Wide Screen for β-Catenin Binding Sites Identifies a Downstream Enhancer Element That Controls c-Myc Gene Expression. Mol Cell Biol. 2008 Dec 15;28(24):7368–79.

4. Yochum GS, McWeeney S, Rajaraman V, Cleland R, Peters S, Goodman RH. Serial analysis of chromatin occupancy identifies β-catenin target genes in colorectal carcinoma cells. Proc Natl Acad Sci. 2007 Feb 27;104(9):3324–9.

5. Tuupanen S, Turunen M, Lehtonen R, Hallikas O, Vanharanta S, Kivioja T, et al. The common colorectal cancer predisposition SNP rs6983267 at chromosome 8q24 confers potential to enhanced Wnt signaling. Nat Genet. 2009 Aug;41(8):885–90.

6. Ahmadiyeh N, Pomerantz MM, Grisanzio C, Herman P, Jia L, Almendro V, et al. 8q24 prostate, breast, and colon cancer risk loci show tissue-specific long-range interaction with MYC. Proc Natl Acad Sci. 2010 May 25;107(21):9742–6.

7. Jäger R, Migliorini G, Henrion M, Kandaswamy R, Speedy HE, Heindl A, et al. Capture Hi-C identifies the chromatin interactome of colorectal cancer risk loci. Nat Commun. 2015 Feb 19;6:6178.

8. Yochum GS. Multiple Wnt/ß-Catenin Responsive Enhancers Align with the MYC Promoter through Long-Range Chromatin Loops. PLOS ONE. 2011 Apr 20;6(4):e18966.

9. Heintzman ND, Hon GC, Hawkins RD, Kheradpour P, Stark A, Harp LF, et al. Histone modifications at human enhancers reflect global cell-type-specific gene expression. Nature. 2009 May;459(7243):108–12.
